# Post-transplant BRAF/MEK inhibitor maintenance sustains complete remission in BRAF V600E-mutant histiocytic sarcoma

**DOI:** 10.1007/s00277-026-07277-6

**Published:** 2026-10-28

**Authors:** Ihab Karim, Tobias Arnold, Andreas Bräuninger, Marius Dohse, Felix Schell, Maisun Abu Samra, Thomas Tarawneh, Andreas Burchert, Stefan Gattenlöhner, Kristina Sohlbach

**Affiliations:** 1 Department of Hematology and Oncology, Carreras Leukämie Centrum, Philipps-Universität Marburg, University Hospital Giessen and Marburg, Marburg, Germany; 2 Department of Hematology and Oncology, University Hospital Giessen and Marburg, Giessen, Germany; 3 Institute of Pathology, University Hospital Giessen and Marburg, Giessen, Germany

**Keywords:** Histiocytic sarcoma, BRAF V600E, Allogeneic stem cell transplantation, Targeted therapy, Maintenance therapy, MEK inhibition

## Abstract

Histiocytic sarcoma is a rare and aggressive malignancy with poor outcomes under conventional chemotherapy. Activating *MAPK* pathway alterations, including *BRAF V600E* mutations, provide a rationale for targeted therapy; however, the durability of responses and the role of post-transplant maintenance remain unclear. We report a patient with refractory *BRAF V600E*-mutant histiocytic sarcoma who achieved complete remission following *BRAF/MEK* inhibition and subsequently underwent allogeneic stem cell transplantation. Post-transplant maintenance therapy with dabrafenib and trametinib was initiated early after engraftment. The patient achieved sustained complete remission with no radiological or molecular evidence of disease during follow-up exceeding one year. Circulating tumor DNA analysis remained negative for previously detected mutations. This case suggests that an integrated strategy combining targeted therapy, allogeneic transplantation, and post-transplant maintenance may enable durable disease control in high-risk *BRAF*-mutant histiocytic sarcoma and warrants further evaluation.

## Introduction

Histiocytic sarcoma (HS) is a rare and aggressive malignancy with limited therapeutic options and poor prognosis in refractory disease [1]. Activating *MAPK* pathway alterations, including *BRAF V600E*, provide a rationale for targeted therapy, and *BRAF*/*MEK* inhibition can induce rapid responses [2]. However, the durability of these responses and their optimal integration with allogeneic stem cell transplantation remain incompletely defined, and the role of post-transplant targeted maintenance has not been systematically addressed. Here, we report a patient with refractory *BRAF V600E*–mutated HS who achieved sustained complete remission following sequential *BRAF*/*MEK* inhibition, allogeneic transplantation, and post-transplant targeted maintenance therapy. Ethics committee approval (No. 25–243 RS) and written informed consent were obtained.

## Case presentation

**Fig. 1:**
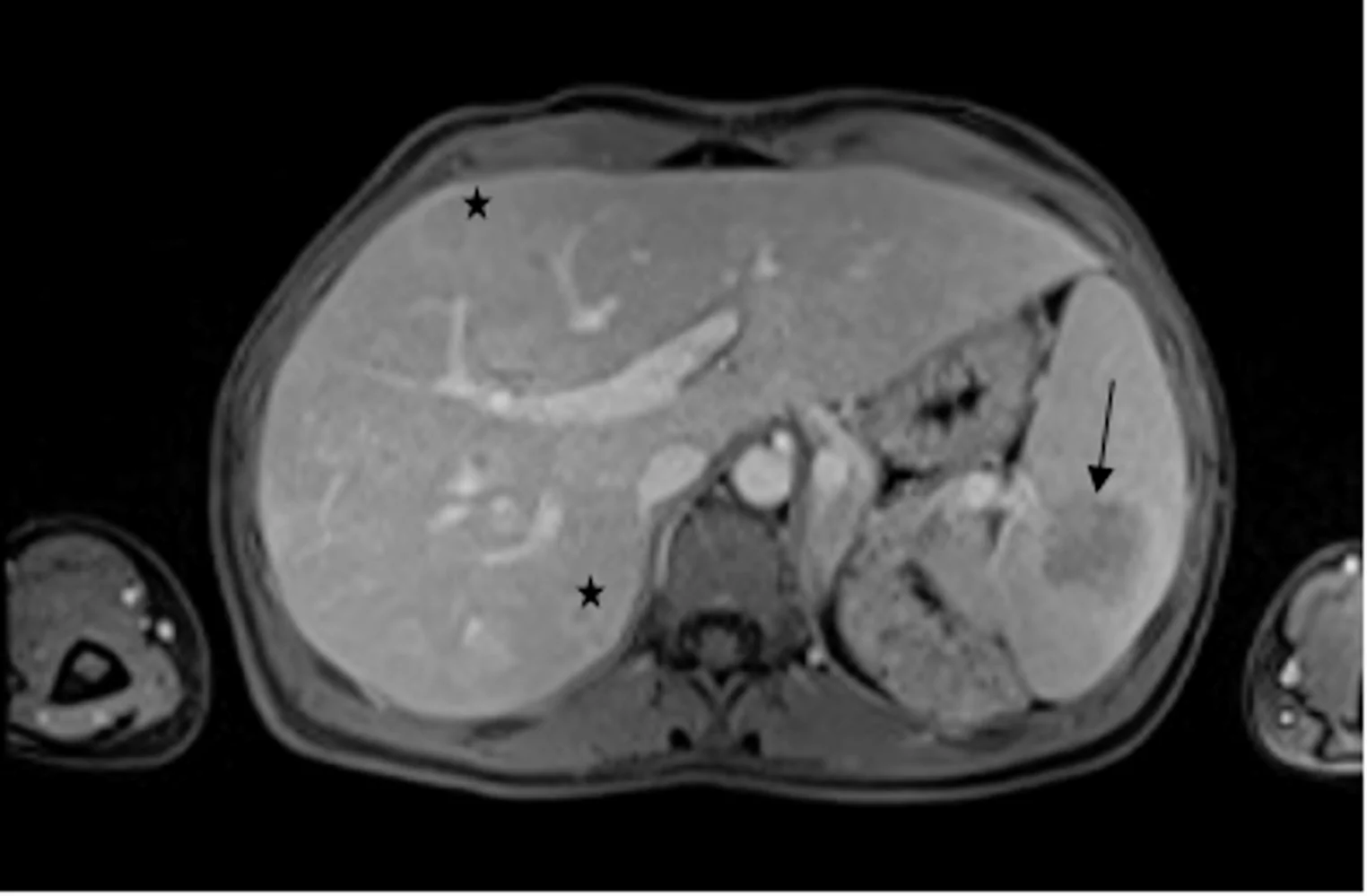
Baseline imaging showing extensive hepatic and splenic involvement. Axial contrast-enhanced magnetic resonance imaging (MRI) of the abdomen demonstrating multiple hypointense lesions throughout both hepatic lobes (*) and a solitary splenic lesion (arrow)

A 35-year-old woman initially presented with fever, fatigue, weight loss (~ 3 kg), and abdominal discomfort. She was febrile (38.8 °C) with hepatosplenomegaly, elevated inflammatory markers, and anemia. Imaging showed multiple hypodense hepatic lesions and a solitary splenic lesion. The patient was transferred to a tertiary care center, where repeat MRI demonstrated rapid progression with the splenic lesion enlarging from 3.7 to 4.8 cm within days (Fig. 1).

A liver biopsy was obtained, and prephase therapy with prednisolone (100 mg/day) was initiated. Subsequent FDG-PET/CT confirmed multiple hypermetabolic hepatic lesions and intense splenic uptake (SUVmax 20.3) without nodal or additional extranodal disease. The patient was initially diagnosed with aggressive lymphoma and received *CHOEP* chemotherapy but showed no response, with radiographic progression.

**Fig. 2:**
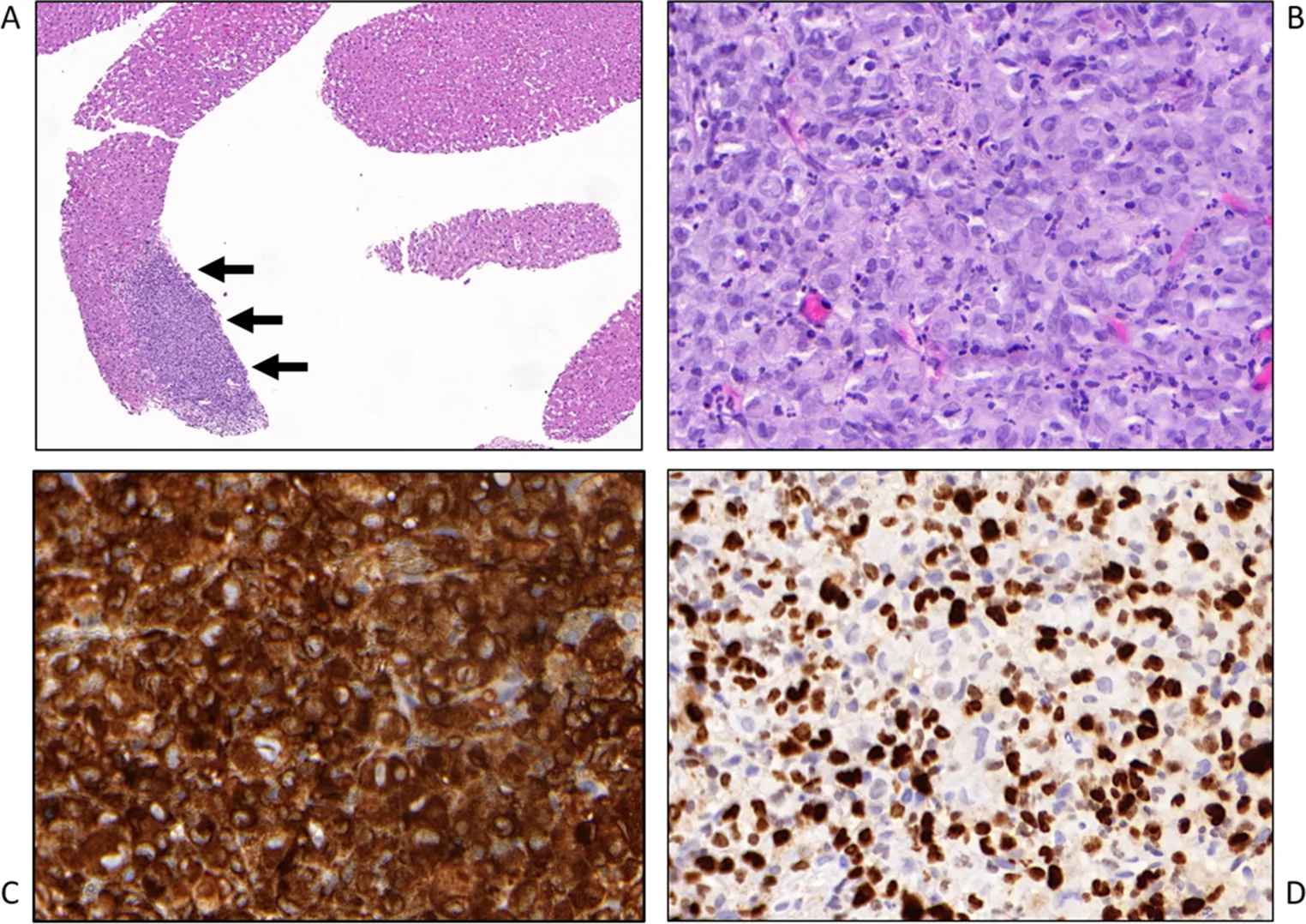
Histopathological and immunohistochemical findings of the diagnostic liver biopsy. (**A**) Histological overview of the diagnostic liver biopsy with focal tumor infiltrates (arrows, H&E, ×20). (**B**) In greater detail, the tumor infiltrates show large tumor cells with pleomorphic nuclei (H&E, ×400). (**C**) Immunohistochemical staining demonstrates strong and specific positivity of the tumor cells for CD68, indicating their histiocytic differentiation (two-step immunoperoxidase reaction, CD68, ×200). (**D**) Ki-67 immunohistochemical staining reveals a high proliferative activity of 60% (two-step immunoperoxidase reaction, Ki-67, ×200)

Histopathological review of liver core biopsies confirmed histiocytic sarcoma (Fig. 2).

Molecular profiling revealed an activating *BRAF V600E* mutation and a *MAP2K1* class 2 (*C121S*) activating mutation (Table 1). Additional molecular findings included a *BCL6* translocation and a clonal immunoglobulin heavy-chain (*IGH*) rearrangement. 

**Table 1:** Molecular alterations identified by targeted next-generation sequencing in the diagnostic liver biopsy specimen, including gene, protein alteration, and variant allele frequency

Gene	Protein alteration	VAF (%)
BRAF	V600E	26.8
MAP2K1	C121S	26.0
CD70	G16R	24.8
DTX1	V27M	24.3
DTX1	N32T	24.7
PLEC	R4166C	50.8
PLEC	A3070V	50.1
TET2	V1949M	44.2
IGLL5	R69G	27.1
BCL10	S141fs	17.0

*VAF* variant allele frequency

Given the chemo-refractory course and *BRAF* (*V600E*) mutation, dabrafenib was initiated, resulting in rapid clinical and radiological improvement. The patient was discharged for outpatient follow-up.

Following the achievement of complete remission with dabrafenib, the patient underwent consolidation with allo-HSCT from a matched unrelated donor, following fludarabine/busulfan-based conditioning. Engraftment was achieved without delay. Early after transplantation, post-transplant maintenance therapy with dabrafenib (2 × 75 mg/day) and trametinib (2 mg/day) was initiated.

**Fig. 3:**
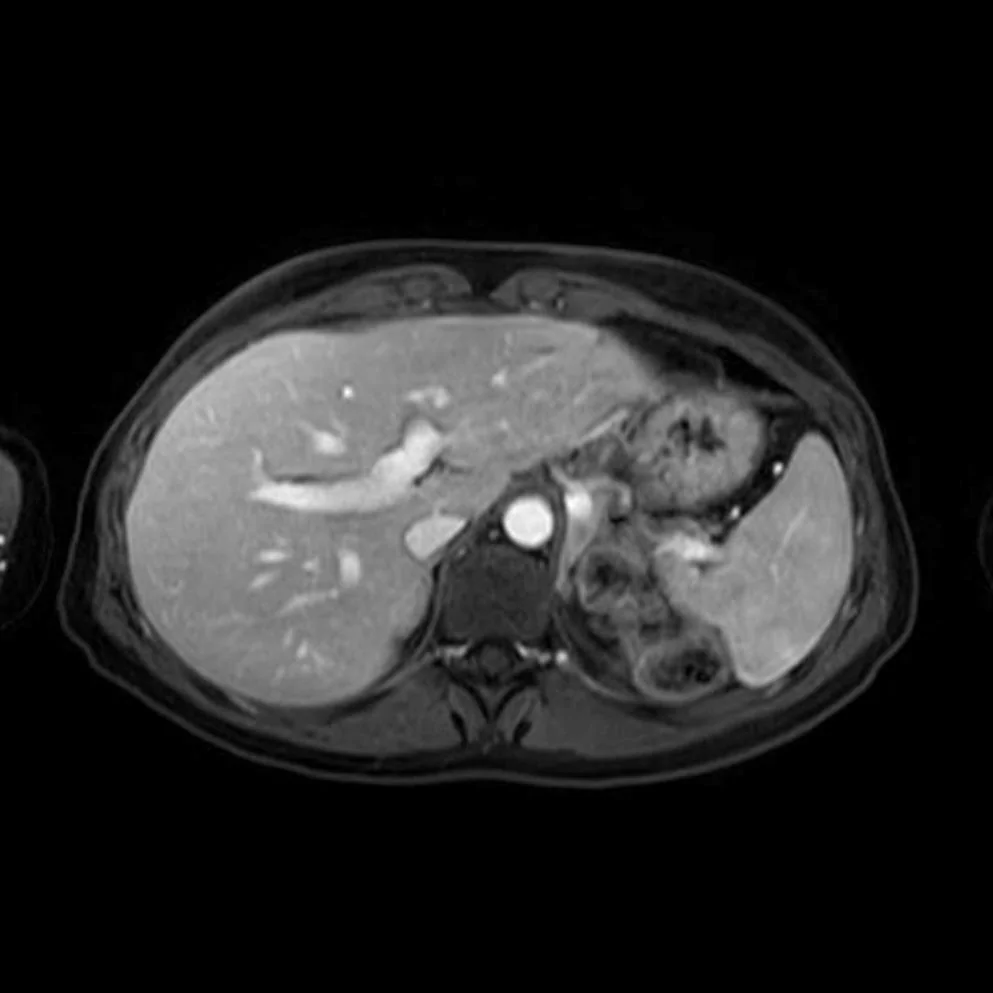
Follow-up imaging after therapy. Axial contrast-enhanced magnetic resonance imaging (MRI) of the abdomen demonstrating normalization of hepatic morphology without identifiable residual focal lesions and marked regression of the splenic lesion, with a small residual scar

Follow-up MRI demonstrated sustained remission with regressed hepatic lesions and a small stable splenic residual (Fig. 3). Twice, ctDNA analysis using targeted NGS detected no previously identified pathogenic variants (BRAF V600E, MAP2K1), consistent with molecular remission.

## Discussion

Histiocytic sarcoma is highly aggressive; population-based data show a median overall survival of only six months with conventional chemotherapy (e.g., *CHOP*, *ICE*, *ABVD*), underscoring the profound chemoresistance observed in our patient who progressed rapidly after *CHOEP* [3].

Diagnosis was challenging, as initial histopathology suggested a high-grade lymphoproliferative disorder, whereas molecular profiling confirmed HS with concurrent *BRAF V600E* and *MAP2K1* class 2 mutation (*C121S*), activating the *MAPK/ERK* pathway, a key oncogenic mechanism in histiocytic neoplasms [4]. Given the chemo-refractory course, treatment with the *BRAF* inhibitor dabrafenib was initiated, leading to rapid metabolic remission, consistent with prior reports [2]. Responses to *MEK* inhibitors such as trametinib have also been reported in HS harboring activating *MAP2K1* mutations [5]. Functional and preclinical evidence, together with clinical observations, support allele-specific *RAF* dependency of *MAP2K1* mutations, with class 2 variants such as *C121S* showing variable sensitivity to *MEK* inhibition [6]. Durable disease control with trametinib has been reported in a patient with a class 2 *MAP2K1*-mutant (*C121S*) stage IV-M1d melanoma [7].

*MAPK*-directed therapy can induce rapid and deep remissions; however, its durability as a standalone treatment appears limited. A large national series of malignant histiocytosis reported a median response duration of approximately seven months and a persistent complete remission rate of 21% with *BRAF*/*MEK* inhibition, markedly lower than outcomes observed in Erdheim-Chester disease (ECD) and Langerhans cell histiocytosis (LCH) treated with targeted therapy alone. In contrast, consolidative allo-HSCT enabled sustained disease control, including ongoing complete remissions beyond 19 months in a subset of patients [8]. These findings suggest that the aggressive biology of these tumors may not be solely dependent on *MAPK* signaling, providing a rationale for consolidative transplantation after initial targeted therapy.

However, the role of post-transplant targeted maintenance has not been systematically addressed. In this context, our case extends existing evidence by suggesting that maintenance therapy may contribute to sustained disease control following transplantation.

The rationale for post-transplant maintenance with dabrafenib/trametinib is multi-faceted. First, this combination has demonstrated high efficacy in related histiocytic disorders such as ECD and LCH [9]. Its potential relevance for HS is further supported by a case report describing stable, long-term disease control with dabrafenib/trametinib in a pediatric patient with secondary HS [10]. Mechanistically, the combination is superior to *BRAF* inhibitor monotherapy in preventing resistance through *MAPK* pathway reactivation, a principle underscored by its proven survival benefit in *BRAF*-mutant metastatic melanoma [11]. Finally, the concept of targeted maintenance after allo-HSCT to suppress minimal residual disease is supported by data in *FLT3-ITD* AML, where *sorafenib* maintenance post-transplant significantly reduces relapse risk [12].

Post-transplant ctDNA analysis detected no residual pathogenic variants. However, liquid biopsy sensitivity in HS is limited by low tumor DNA shedding, particularly in the post-transplant setting; thus, negative results may not reliably exclude residual disease [13].

Post-transplant administration of *BRAF*/*MEK* inhibitors requires careful monitoring but was feasible in our patient without significant toxicity. Although limited by the single-case nature and a follow-up of just over one year, the sustained remission observed here suggests that post-transplant *BRAF*/*MEK* inhibitor maintenance may contribute to durable disease control after allogeneic stem cell transplantation. These findings underscore the importance of routine molecular testing for actionable alterations such as *BRAF* mutations in HS and support further prospective evaluation of integrated strategies combining targeted therapy, allogeneic transplantation, and post-transplant maintenance in high-risk disease. 

## Data Availability

Data are available from the corresponding author upon reasonable request.
